# Exploiting Beneficial *Pseudomonas* spp. for Cannabis Production

**DOI:** 10.3389/fmicb.2021.833172

**Published:** 2022-01-14

**Authors:** Carole Balthazar, David L. Joly, Martin Filion

**Affiliations:** ^1^Department of Biology, Faculty of Sciences, Université de Moncton, Moncton, NB, Canada; ^2^Saint-Jean-sur-Richelieu Research and Development Centre, Agriculture and Agri-Food Canada, Saint-Jean-sur-Richelieu, QC, Canada

**Keywords:** *Cannabis sativa*, marijuana, hemp (*Cannabis sativa* L.), *Pseudomonas*, plant growth-promoting rhizobacteria (PGPR), biological control, abiotic stress, microbial inoculant

## Abstract

Among the oldest domesticated crops, cannabis plants (*Cannabis sativa* L., marijuana and hemp) have been used to produce food, fiber, and drugs for thousands of years. With the ongoing legalization of cannabis in several jurisdictions worldwide, a new high-value market is emerging for the supply of marijuana and hemp products. This creates unprecedented challenges to achieve better yields and environmental sustainability, while lowering production costs. In this review, we discuss the opportunities and challenges pertaining to the use of beneficial *Pseudomonas* spp. bacteria as crop inoculants to improve productivity. The prevalence and diversity of naturally occurring *Pseudomonas* strains within the cannabis microbiome is overviewed, followed by their potential mechanisms involved in plant growth promotion and tolerance to abiotic and biotic stresses. Emphasis is placed on specific aspects relevant for hemp and marijuana crops in various production systems. Finally, factors likely to influence inoculant efficacy are provided, along with strategies to identify promising strains, overcome commercialization bottlenecks, and design adapted formulations. This work aims at supporting the development of the cannabis industry in a sustainable way, by exploiting the many beneficial attributes of *Pseudomonas* spp.

## Introduction

Cannabis (*Cannabis sativa* L.) is an annual dioecious herbaceous plant from the Cannabaceae family. Cannabis crops likely originated from the temperate regions of Eurasia where their early cultivation and domestication began thousands of years ago. Human selection and breeding resulted in the creation of a plethora of cultivated varieties (cultivars) that are now disseminated worldwide (Clarke and Merlin, 2016). Following the steps of early farmers who harvested the stalks, seeds, and inflorescences of wild cannabis plants to produce fiber, oil, and drugs, respectively, generations of breeders have developed modern cultivars with distinctive traits to efficiently produce these economically valuable commodities.

While hemp cultivars harvested for fiber production tend to have a tall stature with elongated stem internodes yielding long bast fibers, oilseed cultivars usually display a short branching architecture producing lots of nutritious seeds (Small, 2015). Fiber and oilseed hemp cultivars must also yield low levels of Δ-9-tetrahydrocannabinol (THC)—the main psychoactive cannabinoid compound of cannabis—to comply with laws in North America and Europe that define hemp as containing up to 0.3% of THC and/or its acidic precursor on a dry weight basis.

On the other hand, marijuana plants have been bred mostly clandestinely for their large female inflorescences (the “buds”) harboring glandular trichomes that yield high levels of psychoactive THC. By eliminating all male plants from the crop to prevent pollination, seedless female inflorescences yielding more than 20% THC by dry weight can thus be obtained from the most potent marijuana cultivars (Clarke and Merlin, 2016). Apart from THC, other molecules of pharmaceutical interest, like cannabidiol (CBD) and aromatic terpenes, can also be obtained from the glandular trichomes of both marijuana and hemp cultivars. These drug-type crops (e.g., marijuana and hemp cultivars grown for phytochemical production, including THC and/or CBD) are very lucrative and are generally reproduced vegetatively by clonal cuttings from elite female plants with desirable genotypes, then transplanted in enclosed outdoor plots, secured greenhouses, or indoor cultivation rooms. Contrastingly, commercial fiber and oilseed hemp crops are mostly directly seeded in agricultural fields for cost efficiency (Small, 2015; Thiessen et al., 2020).

Because of the widespread criminal status associated with some marijuana products, reliable research and economic development with cannabis plants have been largely prevented during most of the 20th century. Therefore, great opportunities and challenges remain to improve cannabis cultivation in the wake of the worldwide legalization trend led by progressive countries like Uruguay and Canada (Taghinasab and Jabaji, 2020).

*Pseudomonas* is a diversified genus of rod-shaped, motile, aerobic Gammaproteobacteria. With more than 200 type strains distributed in intricate lineages and phylogenetic groups, it is currently the Gram-negative genus with the highest number of recognized species (Lalucat et al., 2020). These versatile bacteria are abundant in a wide range of environmental niches, including soil, water, plants, and animals, demonstrating their great metabolic flexibility and lifestyle adaptability. Indeed, while some species are infamous human or plant pathogens, like *Pseudomonas aeruginosa* and *Pseudomonas syringae*, respectively, others like *Pseudomonas putida* and *Pseudomonas fluorescens* have been exploited as bioremediation agents for polluted soils, plant growth-promoting rhizobacteria (PGPR), and biocontrol agents (Sitaraman, 2015). Model organisms for plant-microbe interactions, beneficial *Pseudomonas* spp. are ubiquitous in soils and competitively colonize all compartments of the plant microbiome, including the soil close to the roots (rhizosphere), the surface of aerial organs (phyllosphere), and the inner plant tissues (endosphere). The host plants, in turn, benefit from growth- and health-promoting effects including improved nutrient availability, increased tolerance to abiotic stresses, and repression of pests and diseases by antibiosis, competition, and elicitation of induced systemic resistance (ISR) (Nadeem et al., 2016; Backer et al., 2018). This review aims at identifying important aspects of these beneficial traits to consider when developing *Pseudomonas* spp. inoculants tailored for hemp and marijuana production.

## Occurrence and Diversity of *Pseudomonas* spp. in the Cannabis Microbiome

Many of the numerous studies that started to unravel the cannabis microbiome in recent years have consistently identified *Pseudomonas* spp. as major components of the cannabis rhizosphere, phyllosphere and endosphere communities (Backer et al., 2019; Taghinasab and Jabaji, 2020).

Beneficial species such as *P. fluorescens*, *Pseudomonas protegens*, and *P. putida* seem to be naturally present throughout all hemp and marijuana tissues and surrounding soil compartments (Table 1). For instance, *Pseudomonas* spp. were the most abundant culturable bacteria recovered from above-ground endosphere samples of three different hemp cultivars in Canada, accounting for 44% of all bacterial isolates in leaves, 39% in petioles, and 5% in seeds (Scott et al., 2018). Regarding the below-ground compartments, *Pseudomonas* spp. and related Proteobacteria are also commonly identified as part of the core community of cannabis root colonizers, regardless of the different cropping systems, growing substrates, climatic conditions and host characteristics surveyed at various geographic locations (Table 1).

**TABLE 1:** *Pseudomonas* spp. (or higher related taxa) associated with the cannabis microbiome.

Crop type (cultivar)	Sample type	Environment	Reported taxa	References
Fiber/oilseed hemp (Anka, CRS-1, Yvonne)	Leaves, petioles, seeds (endosphere)	Outdoor field	*Pseudomonas* spp., *Pseudomonas fulva* and *Pseudomonas orientalis*	Scott et al., 2018
Fiber/oilseed hemp (Anka)	Bulk soil, rhizosphere, roots (endosphere), leaves and flowers (phyllosphere)	Outdoor field	*Pseudomonas* spp.	Barnett et al., 2020
Fiber/oilseed hemp (Anka)	Bulk soil, rhizosphere	Indoor growth chamber	*Pseudomonas* spp.	Comeau et al., 2021
Fiber/oilseed/drug hemp (Gansuqingshui, Yunnan 1, Yunmaza 1, and Huoma 1)	Bulk soil, rhizosphere, roots, leaves, stems, flowers (endosphere)	Indoor growth chamber	*Pseudomonas* spp.	Wei et al., 2021
Fiber hemp (Fedora 17)	Retting stems	Outdoor field	*Pseudomonas fluorescens, Pseudomonas psychrotolerans, Pseudomonas rhizosphaerae, Pseudomonas graminis, Pseudomonas fulva, Pseudomonas viridiflava*, and *Pseudomonas syringae*	Ribeiro et al., 2015
Fiber hemp (USO-31)	Retting stems	Outdoor field	*Pseudomonas argentinensis, Pseudomonas rhizosphaera*, and *Pseudomonas syringae*	Liu et al., 2017
Fiber hemp (Futura 75, Felina 32, and SS Alpha)	Retting stems	Greenhouse	*Pseudomonas* spp.	Law et al., 2020
Fiber hemp	Diseased stems	Greenhouse/field	*Pseudomonas syringae*	McPartland and Hillig, 2004
Hemp	Diseased stems or leaves	Natural habitat/field	*Pseudomonas cannabina* and *Pseudomonas syringae*	McPartland et al., 2000
Hemp	Diseased leaves	Outdoor field	*Pseudomonas cannabina*	Bull et al., 2010
Hemp	Bulk soil, rhizosphere	Industrial site	*Pseudomonas balearica* and *Pseudomonas stutzeri*	Liste and Prutz, 2006
Wild hemp	Roots, shoots (endosphere)	Industrial site	*Pseudomonas* sp.	Iqbal et al., 2018
Wild hemp	Rhizosphere, roots (endosphere)	Natural habitat	*Pseudomonas geniculata, Pseudomonas koreensis, Pseudomonas plecoglossicida*, and *Pseudomonas taiwanensis*	Afzal et al., 2015
Drug-type hemp (TJ’s CBD)	Rhizosphere, roots	Outdoor field	Pseudomonadales	Ahmed et al., 2021
Drug-type hemp (Tangerine)	Rhizosphere, roots, leaves, flowers (endosphere)	Outdoor field	Gammaproteobacteria	Willman et al., 2021
Drug-type hemp	Diseased leaves	Outdoor field	*Pseudomonas koreensis*	Thiessen et al., 2020
Drug-type marijuana	Dried inflorescences (medicinal products)	Indoor commercial facility	*Pseudomonas* spp., *Pseudomonas fluorescens, Pseudomonas putida, Pseudomonas stutzeri*, and *Pseudomonas aeruginosa*	McKernan et al., 2016
Drug-type marijuana	Dried inflorescences (medicinal products)	Commercial setting	*Pseudomonas* sp., *Pseudomonas monteilii, Pseudomonas oryzihabitans, Pseudomonas putida, Pseudomonas coleopterorum*, and *Pseudomonas fluorescens*	McKernan et al., 2021
Drug-type marijuana	Dried inflorescences (medicinal products)	Commercial setting	*Pseudomonas fluorescens, Pseudomonas protegens, Pseudomonas putida, Pseudomonas mendocina*, and *Pseudomonas aeruginosa*	Thompson et al., 2017
Drug-type marijuana (CBD Yummy, CBD Shark, and Hash)	Rhizosphere, roots (endosphere)	Indoor commercial facility	Proteobacteria	Comeau et al., 2020
Drug-type marijuana (Sour Diesel, Bookoo Kush, Burmese, Maui Wowie, and White Widow)	Bulk soil, rhizosphere, roots (endosphere)	Commercial setting	*Pseudomonas* spp.	Winston et al., 2014
Drug-type marijuana (Ghost Train, Afgooey, Dulce, Caboose, Special Queen, Gila Kush, Golden Gate, Kandy Kush, Kushy Kush, and Ghost Haze)	Bulk soil, rhizosphere	Greenhouse	*Pseudomonas* spp.	Perea, 2019

As plant genotype and edaphic factors cooperatively shape the structure and diversity of microbial communities within the rhizosphere and root tissues (Bulgarelli et al., 2013), complex mechanisms like modulation of root exudates, root morphology, and regulation of the plant immune system drive the recruitment and proliferation of beneficial microorganisms from surrounding bulk soil toward the roots (Sasse et al., 2018). In this context, since cannabis phytochemicals possess well-documented antimicrobial properties (Ali et al., 2012), they may act as repellents or chemotaxis molecules that potentially inhibit or favor the colonization of bacteria in the vicinity of the roots and/or in the phyllosphere (Comeau et al., 2020). Accordingly, factors like cannabis cultivar and/or developmental stage have been reported to affect the microbiome composition within root tissues (Winston et al., 2014; Comeau et al., 2020), while soil characteristics and cropping practices also exert a preponderant influence on rhizosphere communities (Winston et al., 2014; Perea, 2019; Barnett et al., 2020; Ahmed et al., 2021; Comeau et al., 2021). Based on the ubiquity of *Pseudomonas* spp. in the cannabis microbiome, it is expected that directed microbial inoculations with beneficial strains should be applicable consistently under a wide range of commercial and agricultural conditions.

Phytopathogenic *Pseudomonas* species are also reported on cannabis plants, including *Pseudomonas cannabina* causing bacterial blight (water soaked leaf spots turning into necrotic lesions) (McPartland et al., 2000; Bull et al., 2010) and *P. syringae* pv. *mori* causing striatura ulcerosa (elongated stem lesions with fluid-filled pustules rupturing into ulcers) (McPartland et al., 2000; McPartland and Hillig, 2004). *P. cannabina* and *P. syringae* are host-specialized phyllosphere pathogens that invade the intercellular apoplast space and use a type III secretion system to deliver virulence effectors into the plant cells (Xin et al., 2018). Even though recent reports of bacterial pathogens are surprisingly scarce in cannabis, phytopathogenic *Pseudomonas* spp. are expected to cause emerging disease problems with the surge in hemp and marijuana cultivation (Punja, 2021).

Finally, a last group of *Pseudomonas* strains potentially relevant in cannabis cultivation concerns *P. aeruginosa*. Since this opportunistic human pathogen can cause important infections in immunocompromised and hospitalized patients, screening for contaminations in marijuana products is usually implemented during production quality control and may vary according to local regulations (McPartland and McKernan, 2017). Testing for *P. aeruginosa* contaminants is especially important for fresh raw plant products, since this Gram-negative non-sporulating bacterium is highly sensitive to heat and desiccation, and therefore unlikely to survive the processes of marijuana drying, curing, decarboxylation, extraction and/or smoking (Holmes et al., 2015). Even though little data is available regarding bacterial infections in humans caused by contaminated marijuana (Montoya et al., 2020), *P. aeruginosa* DNA material has still been detected in some medicinal products (McKernan et al., 2016; Thompson et al., 2017) and a severe case of pneumonia has been associated with inhalation of *P. aeruginosa* from a contaminated marijuana smoking device (Kumar et al., 2018). Therefore, preventive precautions should be taken during production to avoid any biosafety risk.

## Modes of Action of Beneficial *Pseudomonas* spp.

**FIGURE 1:**
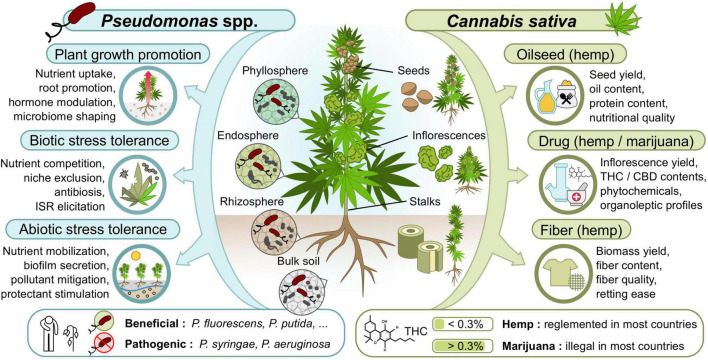
Potential modes of action of beneficial *Pseudomonas* spp. inoculants and traits they could improve in cannabis crops.

Various strategies can be explored to increase the yield and quality of cannabis crops. Several recent literature reviews can be consulted about the current opportunities and challenges associated with cannabis genetic diversity, cultivar breeding and agronomic traits improvement (Salentijn et al., 2015; Clarke and Merlin, 2016; Schluttenhofer and Yuan, 2017; Hesami et al., 2020), cannabinoid elicitation (Gorelick and Bernstein, 2017; Backer et al., 2019), disease management (Punja, 2021), production factors optimization (Backer et al., 2019; Eichhorn Bilodeau et al., 2019), and biosafety practices to reduce contaminants (McPartland and McKernan, 2017; Montoya et al., 2020; Vujanovic et al., 2020). Within all these promising developments, microbiome engineering and beneficial microbial inoculants appear as a recurring prospective trend, potentially promoting plant growth and fitness (Kusari et al., 2017; Backer et al., 2019; Lyu et al., 2019; Söderström, 2020), enhancing cannabinoid production (Lyu et al., 2019; Taghinasab and Jabaji, 2020; Ahmed and Hijri, 2021), controlling diseases (Kusari et al., 2017; Lyu et al., 2019; Söderström, 2020; Punja, 2021), and improving product biosafety (Vujanovic et al., 2020). Research studies have thus started to examine the potential benefits of inoculating beneficial *Pseudomonas* spp. or other microorganisms on cannabis plants (Table 2). The following sections present the modes of action of beneficial *Pseudomonas* spp. in terms of plant growth promotion, product quality, and tolerance to biotic and abiotic stresses, with special emphasis on their relevance for each cannabis crop type (hemp and marijuana supplying fibers, oilseeds, and/or phytochemicals) (Figure 1).

**TABLE 2:** Studies using microbial inoculants with cannabis plants.

Crop type (cultivar)	Inoculant type	Microorganisms inoculated	Experimental setting	Reported effects	References
Fiber/oilseed hemp (Felina 34)	Roots (soil drench)	***Pseudomonas* sp. DSMZ 13134 (Proradix)**	Greenhouse potted plants	Reduced broomrape weed infestation	Gonsior et al., 2004
Fiber/oilseed hemp (Anka)	Roots (soil drench)	***Pseudomonas synxantha* LBUM223, *Pseudomonas simiae* WCS417r**, *Bacillus velezensis* LBUM279, *Bacillus subtilis* LBUM979 (single-strains and consortia)	Indoor growth chamber	Increased plant weight, no ISR, no biocontrol against *Botrytis*	Balthazar et al., 2020
Fiber/oilseed hemp (Anka)	Roots (soil drench)	***Pseudomonas synxantha* LBUM223, *Pseudomonas protegens* LBUM825**, *Bacillus velezensis* LBUM279, LBUM1082, *Bacillus subtilis* LBUM979 (single-strains and consortia)	Indoor growth chamber	Increased plant weight, modulated rhizosphere microbiome	Comeau et al., 2021
Fiber/oilseed hemp (Carmagnola)	Roots (soil mix)	*Glomus mosseae* BEG 12	Outdoor potted plants	Root colonization, better heavy metals translocation, reduced plant weight	Citterio et al., 2005
Fiber/oilseed hemp (Fedora 17 and Felina)	Roots (irrigation system)	*Trichoderma harzianum* T-22 (Trianum-P)	Greenhouse potted plants	Increased root density, plant height, weight, inflorescence yield, CBD content	Kakabouki et al., 2021b
Fiber hemp (USO-31)	Roots (irrigation system)	*Rhizophagus irregularis* (MycoPlant)	Greenhouse hydroponic system	Increased root length, stem weight, quality, P content, seedling survival	Kakabouki et al., 2021a
Oilseed hemp (Finola)	Roots (soil drench)	*Azospirillum brasilense, Gluconacetobacter diazotrophicus, Burkholderia ambifaria, Herbaspirillum seropedicae* (consortium)	Greenhouse potted plants	Root colonization, increased biomass, stem length and weight, cannabinoid, antioxidant, and phenolic contents	Pagnani et al., 2018
Fiber/oilseed hemp (Anka)	Leaves (foliar spray)	***Pseudomonas synxantha* LBUM223, *Pseudomonas protegens* Pf-5**, *Bacillus velezensis* LBUM279, FZB42, LBUM1082, *Bacillus subtilis* LBUM979	Indoor growth chamber	Biocontrol against *Botrytis*	Balthazar et al., 2021
Fiber hemp (YunMa 1)	Leaves (foliar spray)	*Chaetomium* sp., *Fusarium* sp., *Plectosphaerella* sp., *Nigrospora* sp., *Graphium* sp., *Colletotrichum* sp.	Outdoor field	Increased plant growth, antioxidant activity, fiber yield, and/or fiber length	Jin et al., 2014
Fiber hemp (Fibranova)	Retting stems (water incubation)	*Clostridium* sp. L1/6, *Bacillus* sp. ROO40B (consortium)	Indoor water-retting tanks	Increased fiber quality and retting ease	Di Candilo et al., 2010
Fiber hemp (USO-31)	Retting stems (incubation)	*Phlebia radiata* Cel 26	Outdoor dew-retting field	Increased fiber quality and retting ease	Liu et al., 2017
Drug-type hemp	Roots (irrigation system)	***Pseudomonas putida***, *Enterobacter cloacae*, *Citrobacter freundii*, *Comamonas testosteroni* (consortium, Mammoth PTM)	Indoor hydroponic/soil-less systems	Increased inflorescence yield, plant height, stem thickness	Conant et al., 2017
Drug-type marijuana	Rooted stem cuttings	*Trichoderma harzianum*, *Trichoderma asperellum, Gliocladium catenulatum*	Indoor hydroponic system	Stem colonization, biocontrol against *Fusarium*	Punja, 2021
Drug-type marijuana (Afghani Kush, White Rhino)	Inflorescences (post-harvest spray)	*Bacillus amyloliquefaciens* F727 (Stargus), *Gliocladium catenulatum* J1446 (Prestop), *Trichoderma asperellum* T34 (Asperello)	Detached inflorescences	Biocontrol against *Botrytis*	Punja and Ni, 2021
Drug-type marijuana (Copenhagen Kush)	Leaves (foliar spray)	*Streptomyces lydicus* WYEC108 (Actinovate), *Bacillus subtilis* QST713 (Rhapsody), *Bacillus amyloliquefaciens* F727 (Stargus)	Indoor growing room	Biocontrol against powdery mildew	Scott and Punja, 2021

*Pseudomonas spp. highlighted in bold.*

### Plant Growth Promotion by Beneficial *Pseudomonas* spp.

Beneficial *Pseudomonas* spp. and other PGPR employ a variety of plant growth-promoting mechanisms, such as enhancing nutrient availability and modulating phytohormonal balance, that result in increased biomass yield for many crops (Khan et al., 2016; Backer et al., 2018). However, as discussed below, qualitative improvements are equally needed, or even more, by hemp and marijuana producers.

#### Fiber Hemp Crops

In hemp, two main kinds of fibers are derived from the plant stalk, namely the woody xylem fibers (hurd fibers) and the primary phloem fibers (bast fibers). While hurd fibers are valuable by-products with diversified industrial and biofuel uses, the primary bast fibers are the most lucrative commodity and are separated from the rest of the stem tissues by a process called retting. Traditional retting is accomplished by exposing the harvested stems to decaying microorganisms in the field (dew retting), or by immersing the stems in large water tanks (water retting), to degrade pectins and other binding components. The long bast fibers are then extracted from the retted stalks by a mechanical decortication step, and transformed into textiles and other applications (Small, 2015). Fiber hemp cultivars have thus been selected to exhibit key desirable traits, like biomass yield, ease of retting, and bast fiber content, length, and strength (Schluttenhofer and Yuan, 2017). “Dual usage” crops are also commonly employed for both oilseed and fiber production, even though fiber quality is then reduced because of stem lignification during seed maturation (Small, 2015). Cultivars dedicated to specific industrial uses have also been developed with, for example, chlorophyll-deficient stalks for cheaper dyeing and paper pulp processing (Clarke and Merlin, 2016).

Pioneering assays of microbial inoculants in cannabis, using *P. putida*, *P. protegens*, *Pseudomonas synxantha* and *Pseudomonas simiae* among other PGPR microorganisms, have already successfully increased several indicators of biomass and fiber yield, such as total plant weight and/or stem length, diameter, and weight (Jin et al., 2014; Conant et al., 2017; Pagnani et al., 2018; Balthazar et al., 2020; Comeau et al., 2021; Kakabouki et al., 2021a,b). Further studies are needed to assess the full agronomic potentials of this promising avenue with indicators considering fiber quality, such as internode length and bast fiber content (Jin et al., 2014), fiber width (Müssig and Amaducci, 2018), pectin and lignin content reduction and fiber decortication efficiency (Petit et al., 2020). Encouragingly, in other crops like cotton, inoculation with *P. fluorescens* and other PGPR strains effectively increased fiber quality properties and yield (Abdulla and Karademir, 2019). Modulation of phytohormones implicated in phloem differentiation and fiber formation, like auxins, gibberellins and ethylene, has also been demonstrated with *Pseudomonas* spp. (Backer et al., 2018), but the involvement of this hypothetical mechanism remains to be validated in this context. Finally, regarding the retting process, several studies have established that pectinolytic *Pseudomonas* strains are particularly important as natural retting agents for harvested hemp stalks under aerobic conditions (Ribeiro et al., 2015; Liu et al., 2017; Law et al., 2020). Dedicated *Pseudomonas* spp. retting inoculants could therefore be developed to optimize this degradation process which is currently the main factor limiting the quality and production of hemp fibers (Schluttenhofer and Yuan, 2017), as already demonstrated with other microorganisms like fungus *Phlebia radiata* for hemp dew retting (Liu et al., 2017) and bacteria *Bacillus* sp. and *Clostridium* sp. for hemp water retting (Di Candilo et al., 2010).

#### Oilseed Hemp Crops

The oil extracted from hemp seeds is highly nutritious because it is rich in polyunsaturated fatty acids (mostly linoleic acid, α-linolenic acid, oleic acid, and γ-linolenic acid), some of which are essential fatty acids that humans must acquire from their diet. Edible oil can be obtained by cold-pressing the hemp seeds and is added to human food, cosmetics, and industrial fluids, while the residual seed cake can be transformed into protein-rich flour or livestock feed supplements. In countries like Canada, the oilseed hemp industry has even better economic prospects than the fiber market, though it does not currently compete with major oilseed crops like flax, sunflower or canola (Small, 2015).

Significant advances in hemp oil production could be obtained by increasing the seed yield per plant, the seed size, weight and maturity uniformity at harvest, but also its oil and protein contents, and desirable fatty acid and amino acid profiles (Schluttenhofer and Yuan, 2017). However, unlike their tall fiber hemp counterparts, short oilseed cultivars are preferred for an easier mechanized harvest and efficient allocation of the plant energy toward seed production (Small, 2017), while flowering earlier than fiber cultivars allows time for seed maturation during late season. While these quantitative and qualitative improvements are part of ongoing cultivar breeding objectives, the use of *Pseudomonas* spp. inoculants constitutes a promising complimentary approach, as demonstrated in other oilseed crops including sesame (Kumar et al., 2009), sunflower (Majeed et al., 2018), flax (Rajabi-Khamseh et al., 2020), soybean, canola, and corn gromwell (Jiménez et al., 2020) where increased seed yield, oil yield and/or composition in desirable fatty acids were reported. Additionally, more research is still needed to investigate the largely unknown mechanisms resulting in these alterations of seed oil content and composition by PGPR.

#### Drug-Type Marijuana and Hemp Crops

Cannabis phytochemicals accumulate primarily in the secretory cavity of glandular trichomes, which are especially abundant on unpollinated female inflorescences and, to a lesser extent, other aerial plant parts. Over 90 different cannabinoids have been discovered, however, THC and CBD are the most studied because of their psychoactive and therapeutic effects, respectively (Andre et al., 2016). Whereas marijuana cultivars often yield primarily THC and less CBD, hemp cultivars primarily yield CBD and very little THC to comply with legal requirements, as explained above. Consequently, while marijuana crops have found numerous—and sometimes illicit—applications in both the recreational and healthcare markets for a long time, medicinal hemp cultivars with high CBD contents are now of particular interest to the healthcare market as well (Schluttenhofer and Yuan, 2017). Upon harvest, plants are usually dried and cured, then processed into marketable products, including (non-exhaustively) dried inflorescences, resinous preparations (e.g., hashish) and solvent-based extracts, that can either be smoked, inhaled, or ingested (Holmes et al., 2015). While THC and CBD contents mainly drive their market value, the synergistic “entourage effect” of the many other phytochemicals found in plant-based products also contributes to their pharmacological effectiveness compared to single molecules produced in bioreactors (Russo, 2019). These important cannabis secondary metabolites can also alter the organoleptic and qualitative properties of the marketed products, including diverse terpenes conferring unique aroma and flavors, phenolic flavonoids lending antioxidant and anti-inflammatory properties (Andre et al., 2016), and anthocyanin pigments responsible for the purple coloration of popular marijuana cultivars (Small, 2015), among other compounds.

Inoculations with *P. putida* or other beneficial microorganisms have already successfully increased several indicators of phytochemical yield in hemp and marijuana crops, such as inflorescence yield, inflorescence dry weight, and/or cannabinoid content (Conant et al., 2017; Pagnani et al., 2018; Kakabouki et al., 2021b). However, excessive THC elicitation should be avoided in hemp crops, because of the maximum limit permitted by laws for these cultivars. Fortunately, the range of variations in THC content due to environmental factors is quite limited with hemp plants (Small, 2015) and is also restricted by competition with CBD biosynthesis pathways (Jalali et al., 2019). Regarding the qualitative aspects of drug-type crops, increased levels of terpenes, phenols, flavonoids, alkaloids and anthocyanin pigments were obtained with *Pseudomonas* spp. inoculants in other aromatic plants like peppermint, sage, oregano, sweet marjoram, marigold, and geranium; medicinal plants like valerian, datura, black henbane, Madagascar periwinkle, stevia, black *Atractylodes*, turmeric, tea plant, bushmint, and Indian ginseng; and fruit crops like blackberry, strawberry, and pea; as referenced in previous reviews (Bona et al., 2016; Thakur et al., 2019; Çakmakçı et al., 2020). Notably, promotion of phytochemical accumulation by beneficial microorganisms was associated with increased glandular trichome density and/or size in basil, tomato, peppermint, geranium and artemisia (Bona et al., 2016; Çakmakçı et al., 2020; Balestrini et al., 2021).

The mechanisms through which microbial inoculants can alter the accumulation of phytochemicals in aromatic and medicinal plants are not fully understood yet. Hypothetically, beneficial microorganisms may be recognized as a potential threat by the plant which synthetizes phytochemicals as a defense response (Thakur et al., 2019; Balestrini et al., 2021). Supporting this, treatments with stress-related phytohormones significantly increase THC and CBD contents (Mansouri et al., 2009; Jalali et al., 2019; Apicella et al., 2021). Alternatively, promoting root growth and improving nutrient availability and uptake may allow for greater plant biomass and overall wealth, resulting in thriving secondary metabolism functions (Çakmakçı et al., 2020) and early maturation (Conant et al., 2017; Backer et al., 2019). This is supported by the positive response of cannabinoid and inflorescence yields to fertilizer applications during marijuana vegetative growth (Caplan et al., 2017b). Finally, it has been suggested that some endophytes can partially share secondary metabolism pathways with their host and intimately interact with phytochemical production (Ludwig-Müller, 2015), as illustrated by the modulation of terpene metabolism in *Atractylodes* medicinal plants by endophytic *P. fluorescens* (Zhou et al., 2018).

Interestingly, if beneficial microorganisms colonize marijuana inflorescences, they could also potentially influence the marketability of the finished products by directly improving (or deteriorating) their taste and aroma (Winston et al., 2014). Indeed, post-harvest microbiome management is suggested as a valuable application, yet often unexplored, of food-safe microbial inoculants (Berg et al., 2020). This prospect is illustrated by the essential role played by regional microbial communities in defining the unique organoleptic profile (“terroir”) of premium wines, artisan cheeses, and craft beers (Cocolin and Ercolini, 2015). This daring but feasible avenue could be explored by innovative marijuana producers who are noteworthily concerned about the organoleptic distinctiveness of their products (Small, 2015), pending biosafety risk assessments as discussed below. In this context, it should be mentioned that fermenting strains of *Pseudomonas*, especially *P. putida*, are already exploited to synthesize natural flavoring agents like pyrazines (roasted aroma), esters (fruity aroma), vanillin (vanilla aroma), and benzaldehyde (cherry aroma) to alter the organoleptic properties of several food, cosmetic and pharmaceutical products (Bhowmik and Patil, 2018).

### Biotic Stresses and Biocontrol by Beneficial *Pseudomonas* spp.

Biotic stress management is possibly the greatest challenge facing cannabis producers in North America where the recent surge in large-scale cultivation has led to emerging disease outbreaks and increased incidence and severity of pathogens (Punja, 2021). While most hemp and marijuana diseases are caused by fungi and oomycetes, and occasionally by a few bacteria and viruses, other common biotic aggressors include insects, mites, nematodes, weeds, and parasitic plants (McPartland et al., 2000). The prevalence of each disease and pest varies between indoor and outdoor cropping systems (Punja, 2021), but also between fiber, oilseed, and drug-type crops (Bakro et al., 2018; Thiessen et al., 2020).

#### Common Biotic Stresses and Challenges for Their Management

In hemp outdoor production, dense direct-seeded plant stands tend to promote soilborne damping-off pathogens such as *Pythium*, *Thielaviopsis*, *Fusarium*, and *Rhizoctonia* (Thiessen et al., 2020), while various stem-infecting pathogens, including *Fusarium*, *Sclerotinia*, *Phoma*, and *Verticillium*, further reduce oilseed and fiber yields by wilting tissues and collapsing mature plants (McPartland et al., 2000; Bakro et al., 2018). Common outdoor pests include lepidopterous stem borers, beetles, root grubs, caterpillars, leaf miners, seed-eating birds and weeds (McPartland, 1996b). In addition to causing damages to living plants, decaying fungi like *Botrytis*, *Alternaria*, *Trichothecium*, and *Cladosporium* also cause post-harvest quality issues by ruining entire lots of stored seeds (Jian et al., 2019), by spoiling fibers during the retting process (McPartland et al., 2000; Di Candilo et al., 2010), or by releasing mold emissions from finished fiber-based construction materials (Nykter, 2006). Contrastingly, in drug-type crops, inflorescence-infecting pathogens, such as *Botrytis* and *Fusarium*, are often the most damaging with up to 20% of direct yield losses and over 10–15% of post-harvest losses (Punja, 2021). A plethora of other concerning fungal pathogens also thrive in indoor and/or outdoor production, causing leaf spots, blights, mildews, stem cankers and inflorescence rots (McPartland et al., 2000), while saprophytic storage molds like *Aspergillus* and *Penicillium* release mycotoxins and may render finished products unsuitable for human consumption (Montoya et al., 2020). The predominant pests of marijuana plants are spider mites, aphids, whiteflies, mealybugs, and thrips, seemingly bypassing the toxicity of surface cannabinoids thanks to their piercing-sucking mouthparts (McPartland, 1996b). On the other hand, weeds and parasitic plants are not as relevant in well managed greenhouses and cultivation rooms as they are in open fields.

In both hemp and marijuana crops, managing biotic stresses is especially challenging because of the limited range of registered pesticides available (Punja, 2021), the lack of formal agricultural recommendations and mitigation strategies based on reliable research (Sandler and Gibson, 2019), the high susceptibility of modern cultivars to fungal pathogens (Clarke and Merlin, 2016), and the regional variabilities in pest and disease pressures (Thiessen et al., 2020). Additionally, for drug-type crops, finished products destined to human consumption must comply with strict regulations on pesticide residues and microbial contaminants (McPartland and McKernan, 2017). Therefore, in addition to good management practices and crop resistance breeding efforts reviewed recently (Punja, 2021), inoculation with beneficial *Pseudomonas* spp. have been proposed to help cannabis plants cope with biotic stresses (Lyu et al., 2019; Söderström, 2020). Promising research avenues can be loosely sorted into two categories based on a local or systemic mode of action of the inoculants, as detailed below.

#### Local Effects of *Pseudomonas* spp. Inoculants

By colonizing soil niches and plant tissues and producing biofilms, beneficial *Pseudomonas* spp. can compete for space and nutrients, shape microbiome communities, and locally inhibit the growth of pathogens by antibiosis. A plethora of cell wall-degrading enzymes (extracellular chitinases, cellulases, β-1,3-glucanases, proteases, lipases, etc.), antibiotics (pyrrolnitrin, pyoluteorin, phenazines, 2,4-diacetyl phloroglucinol DAPG, etc.), siderophores (pyoverdine, pseudomonine, pyochelin, etc.) and other antimicrobial compounds (including volatile organic compounds like hydrogen cyanide HCN) produced by *Pseudomonas* strains have been characterized (Fischer et al., 2013; Khan et al., 2016; Backer et al., 2018). Notably, their antagonistic activities can be used to control fungal and bacterial diseases, but also herbivorous insects (Flury et al., 2016; Khan et al., 2016), parasitic nematodes (Trivedi and Malhotra, 2013) and weeds (Trognitz et al., 2016). Accordingly, antibiotic production was suggested as the main antifungal mechanism of beneficial *P. protegens* and *P. synxantha* strains when sprayed on hemp leaves to control *Botrytis* gray mold, and/or inhibiting the *in vitro* growth of *Botrytis*, *Sclerotinia*, *Fusarium*, *Alternaria*, and *Phoma* (Balthazar et al., 2021). Previous studies also demonstrated that two strains of *Pseudomonas koreensis* and *Pseudomonas taiwanensis* found in the hemp rhizosphere could inhibit the growth of *Aspergillus* and/or *Fusarium in vitro* by producing siderophores, cellulases, pectinases and proteases (Afzal et al., 2015), while hemp endophytes *Pseudomonas fulva* and *Pseudomonas orientalis* inhibited the growth of *Botrytis, Sclerotinia* and/or *Rhizoctonia in vitro* by producing siderophores, cellulases, and antibiotics (Scott et al., 2018). However, these results were not validated on plants (Afzal et al., 2015; Scott et al., 2018). Ongoing research with other beneficial bacteria and fungi, like *Bacillus*, *Streptomyces*, *Trichoderma*, and/or *Gliocladium*, further supports the potential of antagonistic biocontrol agents against *Botrytis* on marijuana inflorescences (Punja and Ni, 2021) and hemp leaves (Balthazar et al., 2021), and against *Fusarium* and powdery mildew on marijuana stem cuttings and leaves, respectively (Punja, 2021; Scott and Punja, 2021). Besides, various fungal endophytes were also isolated from hemp and marijuana plants previously, but their antimicrobial activities were only tested *in vitro* (Gautam et al., 2013; Kusari et al., 2013; Qadri et al., 2013). Guidelines for timing of application are also important to establish as curative effects are usually harder to achieve than preventive protection (Balthazar et al., 2021; Punja and Ni, 2021).

#### Systemic Effects of *Pseudomonas* spp. Inoculants

Regarding their second mode of action, many rhizospheric *Pseudomonas* spp. are known to trigger a systemic state of alert in their host plant during root colonization, which activates the plant defenses against a broad spectrum of pathogens, viruses, and herbivorous insects. This immune response, acting beyond the site of inoculation and enhancing the defensive capacity of the entire plant, is called Induced Systemic Resistance (ISR) (Pieterse et al., 2014). In prevision of pathogen attacks, plants primed with ISR may exhibit faster and/or stronger expression of basal defense responses such as deposition of callose, lignin, and phenolic compounds, increased activities of chitinase, peroxidase, and phenylalanine ammonia lyase, production of phytoalexins and primed expression of stress-related genes (Fischer et al., 2013). While potential molecular processes and phytohormonal signaling have been investigated, the exact ISR triggers and onset mechanisms remain largely unknown and seem to depend on the specificity of the mutual interactions between certain plants, bacteria and pathogens (Beneduzi et al., 2012). In hemp, an early study suggested that ISR responses were triggered by drenching the soil with a *Pseudomonas* strain, thereby reducing infestations of *Orobanche ramosa* (broomrape parasitic weed) by 80%. However, this presumed mechanism was only hypothetical and not experimentally validated (Gonsior et al., 2004). Conversely, a recent study concluded that drenching the soil with *P. synxantha*, *P. simiae* and/or *Bacillus* spp. rhizobacteria did not protect hemp against *Botrytis* foliar infection and failed to trigger defense-related gene expression in hemp leaves (Balthazar et al., 2020). This unsuccessful attempt was tentatively attributed to a lack of production of ISR-inducing compounds by the bacteria, or to the inability of hemp to perceive such compounds in the rhizosphere (Beneduzi et al., 2012). As ISR interactions are notoriously complex, many possibilities remain to be explored regarding this intriguing mode of action of *Pseudomonas* spp. inoculants.

### Abiotic Stresses Tolerance With Beneficial *Pseudomonas* spp.

Unfavorable environmental factors like salinity, drought, heat or cold, soil pollutants, or nutrient deficiencies are known to impact cannabis physiology and development, and to predispose crops to diseases (McPartland, 1996a). In the context of global climate change and growing human population, abiotic stresses are expected to take an increasing toll on agricultural production worldwide (Camaille et al., 2021). Unfortunately, as with most modern crops, domesticated cannabis cultivars tend to have narrower tolerances to stressful environments compared to their wild counterparts (Small, 2015). In this context, beneficial *Pseudomonas* spp. inoculants may be advantageous to promote cannabis growth under adverse conditions and/or expand its ecological range.

#### Salinity, Water, and Temperature Stresses

Cannabis plants do not tolerate excessive salt (NaCl) in soil, nor brackish waters and salty breezes which can stunt their growth in coastal environments (McPartland and McKernan, 2017). Additionally, in irrigated and indoor production settings, over-fertilization (Caplan et al., 2017a) or the use of water softening systems (McPartland et al., 2000) can also lead to sodium accumulation in the root zone. Cultivar-specific adaptative responses are being investigated in hope of breeding hemp cultivars with broader tolerance to saline conditions (Liu et al., 2016). Regarding water stresses, exposure to drought causes cannabis plants to wilt and predisposes them to fungal canker diseases (McPartland, 1996a), whereas overwatering and flooding also cause wilting (McPartland et al., 2000) but favor root and crown rot diseases in indoor production systems (Punja, 2021). Air humidity further promotes the development of bud rot pathogens within the compact moisture-retaining inflorescences of drug-type cultivars (Clarke and Merlin, 2016), while soil water content and salinity have significant impacts on the rhizosphere microbiome structure (Winston et al., 2014). Field-grown hemp crops can also be significantly damaged by excessive rainfalls and waterlogging (Thiessen et al., 2020) or, conversely, by water shortage and hot temperature in semi-arid environments (Cosentino et al., 2013). However, field-grown hemp generally withstands drought periods thanks to a long taproot allowing access to deep water sources (Small, 2015). Unfortunately, in contrast to fiber and oilseed crops that are direct-seeded, most mass-produced drug-type plants are propagated vegetatively and therefore do not develop taproots (Jin et al., 2021). When transplanted outdoors, these clones commonly exhibit root binding issues (poor root development) and suffer from inadequate water supply (Thiessen et al., 2020). Additionally, since gradual drought stress can be deliberately applied to container-grown cannabis plants to maximize inflorescence and cannabinoid yield (Caplan et al., 2019), drug-type crops under controlled horticultural management may also experience dehydration stress. Finally, regarding adaptation to temperatures, hemp plants are usually better adapted to cool temperate climates than marijuana cultivars originating from hot semi-tropical regions (Small, 2017), even though cultivar-specific differences are reported (Cosentino et al., 2013; Mayer et al., 2015). Hemp seeds still require rather elevated temperatures to germinate, and oilseed cultivars also require a warmer and longer season than fiber crops to allow seed maturation (Small, 2017). While brief exposure to freezing temperatures can be tolerated by most hemp seedlings and mature plants (Mayer et al., 2015; Small, 2017), frosted inflorescences of drug-type crops can turn black and develop a harsh taste compromising their market value (McPartland et al., 2000).

Owing to their great metabolic versatility, *Pseudomonas* spp. can maintain their growth in stressful environments where they can also help plants cope with various abiotic stresses (Nadeem et al., 2016). Successful reduction of salinity stress has been reported in maize, rice, wheat, soybean, alfalfa, bean, tomato, and radish inoculated with halotolerant *Pseudomonas* strains, as referenced in previous reviews (Nadeem et al., 2016; Khan et al., 2019; Bhat et al., 2020). Frequently reported mechanisms of action include the improvement of water and nutrient uptake by roots, upregulation of plant osmoprotectants (proline and glycine betaine) and antioxidant activities (catalase, peroxidase, superoxide dismutase, etc.), maintenance of ionic homeostasis within plant tissues (sodium to potassium ratio), modulation of stress-induced phytohormones (abscisic acid, ethylene, auxins, etc.), and secretion of exopolysaccharide biofilms that trap sodium cations in excess in the rhizosphere (Piccoli and Bottini, 2013; Nadeem et al., 2016; Bhat et al., 2020). In particular, endophytic bacteria with enzymatic 1-aminocyclopropane-1-carboxylate (ACC) deaminase activity can reduce the production of stress-induced ethylene in the roots, thus suppressing its adverse effect on plant growth (Backer et al., 2018). Interestingly, a salt-tolerant *Pseudomonas geniculata* endophyte has been isolated from hemp and promoted canola growth under salinity stress, even though this effect was not validated toward hemp growth (Afzal et al., 2015). Similarly, *Pseudomonas*-induced tolerance to drought or flood has been reported in other plants than cannabis, including maize, rice, wheat, sunflower, pea, mung bean, chickpea, Aleppo pine, and *Arabidopsis thaliana*, as referenced in previous reviews (Liu and Zhang, 2015; Nadeem et al., 2016; Backer et al., 2018; Kour et al., 2019; Camaille et al., 2021). Reported mechanisms of action included stomatal closure preventing water loss, modification of root length and architecture increasing soil exploration and water access, protection of membrane integrity, secretion of exopolysaccharide biofilm improving soil aggregation and water retention, as well as several of the mechanisms presented above (regulation of osmoprotectants, antioxidants, phytohormones, etc.) since osmotic and oxidative stresses are experienced by plants in both dry and saline environments (Nadeem et al., 2016; Kour et al., 2019; Camaille et al., 2021). Regarding exposure to unfavorable temperatures, inoculation with psychrotolerant *Pseudomonas* spp. increased cold tolerance of canola, lentil, mung bean, wheat, and tomato (Nadeem et al., 2016; Yadav et al., 2019), while thermotolerant strains improved tolerance to elevated temperatures in wheat, sorghum, chickpea, and potato (Nadeem et al., 2016; Singh et al., 2019). Reported mechanisms included the production of antifreeze proteins and biofilms, protection of membrane integrity, modulation of phytohormones and antioxidants, improved nutrient acquisition, and increased plant metabolite levels (Nadeem et al., 2016; Yadav et al., 2019). Additionally, foliar applications of *P. fluorescens* could prevent frost injuries on pear trees by competing against ice-nucleating *P. syringae* pathogens that trigger ice crystals formation at low temperatures to breach plant tissues (Lindow, 1983). Interestingly, ice-nucleating abilities have been identified for cannabis pathogen *P. cannabina* (Bull et al., 2010; Xin et al., 2018), suggesting that foliar applications of antagonistic bacteria could also potentially protect hemp and marijuana crops against pathogen-triggered frost injuries.

#### Soil Pollutants and Bioremediation

In the wake of industrial and agricultural intensification, anthropogenic activities have led to the accumulation of concerning pollutants in the environment, like heavy metals, pesticides, and petroleum hydrocarbons. Soil bioremediation is a process that uses microorganisms and plants to detoxify these contaminants and restore soil health in an eco-friendly manner (Kahlon, 2016). Inheriting from its weedy ancestors the ability to thrive in human-disturbed habitats (Small, 2015), hemp is one of the most investigated plants in phytoremediation and exhibits a great capacity to accumulate pollutants in its tissues (Wu et al., 2021). Benefiting from a short life cycle, large root system, and high biomass yield, hemp has been widely used to decontaminate industrial wastewaters and polluted soils from metals like cadmium, copper, nickel, zinc, lead, selenium, cobalt; organic contaminants like benzo[a]pyrene, naphthalene, chrysene and petrol hydrocarbons; and radioactive isotopes of cesium and strontium (McPartland and McKernan, 2017; Wu et al., 2021). While numerous non-food applications are available for the disposal of harvested plant parts, like biofuels and fiber-based construction materials (Wu et al., 2021), seeds containing high levels of particular elements that are valuable for human consumption, such as selenium, can also be used as dietary supplements and biofortified nutraceuticals, thus offering additional application prospects (Stonehouse et al., 2020). On the other hand, contrasting with their ability to accumulate metals and hydrocarbons, cannabis plants are very susceptible to herbicide injuries caused by agrochemicals like clomazone and paraquat that are carried over by air drift and water runoff from adjacent fields (McPartland et al., 2000), or by soil residues from previous land use (Thiessen et al., 2020).

While these compounds are translocated within hemp tissues to be detoxified and/or sequestrated, associated phytotoxic effects might damage plants grown on contaminated soil, causing stunted growth, reduced seed germination (Liste and Prutz, 2006), and chlorosis (Thiessen et al., 2020). Such deleterious effects can be mitigated by beneficial rhizobacteria like *Pseudomonas* spp. which contribute to *ex planta* biodegradation and sequestration processes in the rhizosphere, thereby reducing the contaminant load beforehand (Wu et al., 2021). For example, hemp plants growing in soil from a former manufactured gas plant were found to recruit specific *Pseudomonas* spp. that could degrade polycyclic aromatic hydrocarbons in the soil, thus reducing their phytotoxic effects (Liste and Prutz, 2006). Similarly, hemp plants irrigated by wastewaters from an oil refinery harbored endophytic *Pseudomonas* spp. with phenol and benzene degradation activities, which could be developed into bacterial inoculants to accelerate hydrocarbon phytoremediation (Iqbal et al., 2018). Inoculation of hemp with mycorrhizal fungi also significantly enhanced the translocation of toxic metals from roots to shoots, thus improving the collection of contaminated plant parts and the phytoextraction efficiency of hemp (Citterio et al., 2005). In other crops, beneficial *Pseudomonas* spp. have been reported to degrade and/or reduce the phytotoxic effects of herbicides 2,4-D, glyphosate, atrazine, quizalofop-*p*-ethyl, and clodinafop, insecticides DDT, aldrin, lindane, fipronil and pyriproxyfen, fungicide tebuconazole, and hazardous chemicals trichloroethylene, anthracene, naphthalene, and phenanthrene, among many other aromatic and chlorinated compounds (Kahlon, 2016; Nadeem et al., 2016; Trognitz et al., 2016). Similarly, increased plant tolerance to various heavy metals is mediated by *Pseudomonas* spp. rhizobacteria through extracellular chelation, rhizosphere acidification, redox transformation, intracellular sequestration, and modulation of plant oxidative stress and ethylene production (Nadeem et al., 2016; Lata et al., 2018; Khan et al., 2019).

These promising results could play a central role in enhancing both the range of contaminants that can be remediated with hemp and the rate of their degradation. However, in oilseed and drug-type crops, as harmful contaminant accumulation in consumed seeds and inflorescences can cause human health issues, maximum concentration levels are mandated by laws for pesticides, carcinogenic hydrocarbons, and several heavy metals (Mihoc et al., 2012; McPartland and McKernan, 2017; Montoya et al., 2020). Therefore, for these crops, the use of bacterial inoculants that would enhance the translocation of pollutants from roots to shoots should be avoided, with a potential exception for biofortified seed crops mentioned above. Nevertheless, inoculants with other modes of action, like *ex planta* degradation and below-ground sequestration, could still be advantageous, especially since fertilizers and rockwool media used in marijuana hydroponic systems are particularly vulnerable to heavy metal contamination (McPartland and McKernan, 2017).

#### Nutrient Deficiencies

Fertilization is one of the most important factors in indoor marijuana production and several recent studies have sought to determine the optimal nutrient application rates and timing to achieve high yields and marketable phytochemical profiles (Caplan et al., 2017a,b; Saloner and Bernstein, 2021; Shiponi and Bernstein, 2021). Moreover, soil nitrogen content has a strong structuring effect on the rhizosphere microbiome communities across distinct marijuana cultivars (Winston et al., 2014). Hemp plants also respond with significantly increased growth to adequate soil nutrient supply (Vera et al., 2010; Small, 2015; Deng et al., 2019), yet the lack of updated fertilization recommendations is commonly reported as an important crop management limitation by hemp producers (Thiessen et al., 2020). The most common deficiencies for cannabis usually arise from shortages of macronutrients (nitrogen N, phosphorus P, potassium K, calcium Ca, magnesium Mg, and sulfur S), while micronutrients (zinc Zn, manganese Mn, iron Fe, copper Cu, boron B, chlorine Cl, and molybdenum Mo) are also essential in minute quantities and in excess may cause phytotoxicity (McPartland et al., 2000; Thiessen et al., 2020). Compared to fiber hemp, oilseed and drug-type crops require about as much of soil nitrogen, less potassium but more phosphorus for flowering or seed production (McPartland et al., 2000). Other elements like magnesium, iron, and manganese are also involved in cannabinoid biosynthesis regulation (McPartland et al., 2000) and oilseed production (Mihoc et al., 2012). Accordingly, optimal fertilization during marijuana vegetative stage allows for larger plants resulting in increased inflorescence weight, higher THC content, and potentially more frequent crop turnover due to reduced maturation time (Caplan et al., 2017b). However, fertilization during marijuana flowering stage usually leads to a diluting effect on phytochemical yield, meaning that increasing the biomass of inflorescences lowers their cannabinoid concentration, even though the total cannabinoid production may still increase (Caplan et al., 2017a; Saloner and Bernstein, 2021; Shiponi and Bernstein, 2021).

As introduced above, soil microbial communities can promote plant growth through a variety of mechanisms, including improving the availability of nutrients and their acquisition by plants (Backer et al., 2018). Plant growth-promoting *Pseudomonas* spp. thus hold great potential to help cannabis crops cope with nutrient deficiencies (Lyu et al., 2019; Söderström, 2020). For example, diazotrophic *Pseudomonas stutzeri* strains can fix atmospheric nitrogen gas into biologically usable forms like ammonia, therefore increasing nitrogen available to plants like Indian grass and rice (Khan et al., 2016). Many rhizospheric *Pseudomonas* strains also synthesize low amounts of phytohormonal indole-3-acetic acid (IAA, auxin) and/or decrease growth-inhibiting ethylene production in plant tissues via ACC deaminase activity. These phytohormonal modulations stimulate the development of lateral and adventitious roots and improve soil exploration and nutrient uptake by wheat, maize, canola, tomato (Patten and Glick, 2002; Nadeem et al., 2016), and many medicinal and aromatic plants (Çakmakçı et al., 2020). Treating seeds with root-promoting *Pseudomonas* spp. also improves germination and seedling survival in canola (Patten and Glick, 2002), maize, oilseed rape, sorghum, and sugar beet (Zachow et al., 2013), which could be beneficial for seeded crops of fiber and oilseed hemp. Root-promoting *Pseudomonas* spp. also improve the rooting success of vegetative cuttings in mint (Kaymak et al., 2008), mung bean (Mayak et al., 1999; Patten and Glick, 2002), ficus (Sezen et al., 2014), eucalyptus (Teixeira et al., 2007), and blackcurrant (Dubeikovsky et al., 1993). As mentioned above, rooting success is very important for drug-type cannabis clones that are mass-produced by vegetative cuttings (Thiessen et al., 2020). However, preliminary assays with PGPR inoculants in commercial settings did not substantially improve root emergence on marijuana cuttings (Conant et al., 2017). Finally, *Pseudomonas* spp. rhizobacteria secrete organic acids, HCN, phosphatases, phytases and siderophores that help release important nutrients from their non-labile forms in soil (Backer et al., 2018). Such mineralization and/or solubilization processes are mediated by enzymatic activities, chelating substances, and rhizosphere acidification, and can increase the bioavailability of phosphorus (Rathinasabapathi et al., 2018; Kalayu, 2019), iron (Lemanceau et al., 2009), potassium (Kour et al., 2020), and zinc (Kumar et al., 2019). Interestingly, in hemp, the endophytes *P. geniculata* and *P. koreensis* were found to produce siderophores, IAA, and phosphorus-solubilizing compounds, and to promote canola root development *in vitro*. However, these beneficial traits were not further validated toward hemp growth (Afzal et al., 2015). Other studies reported that a consortium of phosphorus-solubilizing bacteria—including *P. putida* (Baas et al., 2016)—significantly increased marijuana inflorescence yield by 16.5% and promoted faster plant maturation under commercial production (Conant et al., 2017), while a consortium of four diazotrophic bacteria—other than *Pseudomonas* spp.—promoted hemp growth comparably to a conventional nitrogen fertilizer (Pagnani et al., 2018). Nutrient-solubilizing or mycorrhizal fungi like *Trichoderma*, *Glomus*, and *Rhizophagus* also influenced hemp growth, phosphorus uptake and/or seedling quality (Citterio et al., 2005; Kakabouki et al., 2021a,b). Additional studies reported positive effects of *Pseudomonas* spp. inoculants on hemp growth but without investigating the underlying mechanisms (Balthazar et al., 2020; Comeau et al., 2021).

## Strategies to Identify Promising *Pseudomonas* spp.

**FIGURE 2:**
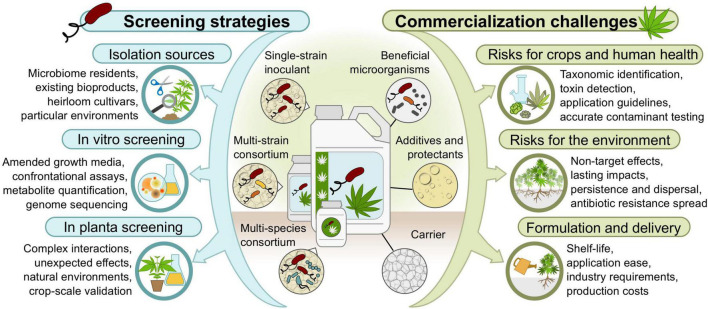
Strategies and bottlenecks to identify beneficial *Pseudomonas* strains and develop bioproducts for cannabis crops.

The many promising avenues presented above suggest that *Pseudomonas* spp. inoculants with multiple modes of action could be developed for use in integrated management of cannabis crops to promote yield and harvest quality under various biotic and abiotic stresses. The following sections explore where to find *Pseudomonas* strains with such beneficial attributes, and suggest screening strategies to assess their abilities *in vitro*, *in planta*, and in consortia (Figure 2).

### Where to Look for Beneficial *Pseudomonas* spp.

Two main strategies are usually distinguished when selecting beneficial microorganisms for plant inoculation and microbiome engineering: the bottom-up or synthetic approach which pieces together candidates exhibiting desired traits from an extensive pool gathered from various sources, and the top-down or stepwise approach, which starts from a complex existing microbial community and identifies its keystone players (Tabacchioni et al., 2021). In cannabis, bottom-up studies seem undeniably promising given the recent results obtained with existing PGPR strains (Citterio et al., 2005; Jin et al., 2014; Pagnani et al., 2018; Comeau et al., 2021), biocontrol agents (Balthazar et al., 2020, 2021), or commercialized bioproducts (Gonsior et al., 2004; Conant et al., 2017; Kakabouki et al., 2021a,b; Punja, 2021; Punja and Ni, 2021; Scott and Punja, 2021), thus paving the way for future work with existing *Pseudomonas*-based bioproducts that are already registered for cereals, fruit trees, and greenhouse vegetables (Fischer et al., 2013; Khan et al., 2016). On the other hand, several top-down studies have also started to examine the potential of harnessing cannabis microbiome residents to improve pathogen control (Gautam et al., 2013; Kusari et al., 2013; Qadri et al., 2013; Scott et al., 2018; Ahmed et al., 2021), salinity tolerance (Afzal et al., 2015), soil phytoremediation (Liste and Prutz, 2006; Iqbal et al., 2018), cannabinoid production (Ahmed and Hijri, 2021), and fiber retting process (Di Candilo et al., 2010; Ribeiro et al., 2015; Liu et al., 2017; Law et al., 2020). In this regard, exploring the microbiome of wild cannabis plants and ancestral heirloom cultivars within their native Eurasian habitats could be of crucial importance to identify beneficial microorganisms that have associated with cannabis over long evolutionary periods. Indeed, such microorganisms were likely lost during the domestication process, which often reduces the variety of plant-associated microbes compared to geographic areas with higher biodiversity on the plant side (Ludwig-Müller, 2015; Berg and Raaijmakers, 2018). This unintended process may even be exacerbated in cannabis grown indoors because of the strict sanitation measures and repeated establishment of microbe-free planting materials (Punja, 2021), inadvertently filtering out beneficial organisms in highly domesticated cultivars. Alternatively, beneficial bacteria could also be sourced from environments where a specific benefit is observed, such as biocontrol strains typically found in disease suppressive soils (Pieterse et al., 2014), plant growth-promoting bacteria isolated from rich undisturbed forest soils (Ahmed and Hijri, 2021; Pirttilä et al., 2021), microorganisms altering organoleptic properties of food products (Cocolin and Ercolini, 2015; Bhowmik and Patil, 2018), and abiotic stress-ameliorating microbes retrieved from extreme environments like dry, hypersaline, cold, or geothermal sites (Zachow et al., 2013; Backer et al., 2018; Pirttilä et al., 2021).

### *In vitro* Screening for Promising *Pseudomonas* spp.

Specific *in vitro* assays are commonly used to quickly screen promising microorganisms for their plant growth-promoting characteristics. For examples, phosphate solubilization abilities can be assessed by growing bacteria on Pikovskaya’s (PVK) agar, nitrogen fixation by using N-free medium, siderophore production with chrome azurol S (CAS) agar, IAA and other phytohormones production with Salkowski reagent and colorimetric methods, ACC deaminase activity with Dworkin and Foster’s (DF) salts medium (Afzal et al., 2015; Ngalimat et al., 2021), biofilm formation with crystal violet staining (Selin et al., 2009), and metabolic capabilities with Biolog microarrays (Gómez-Lama Cabanás et al., 2018; Zboralski et al., 2020). For screening of biocontrol determinants, *in vitro* confrontational assays are commonly used to assess the growth inhibition of culturable pathogens (Afzal et al., 2015; Kusari et al., 2017; Balthazar et al., 2021), Mueller Hinton (MH) media are used for antibiotic diffusion assays, Cyantesmo paper for HCN production, UV-visible spectroscopy for phenazines and pyoverdine detection, and gas and/or liquid chromatography methods for quantification of volatile compounds (VOCs) and soluble antibiotics (Selin et al., 2009; Gómez-Lama Cabanás et al., 2018; Ngalimat et al., 2021). Additionally, lytic enzyme activities like proteases, pectinases and chitinases, can be detected on minimal media amended, respectively, with skim milk, pectin, and colloidal chitin (Afzal et al., 2015; Gómez-Lama Cabanás et al., 2018). Finally, tolerance to abiotic stresses such as salinity, drought and soil pollutants can be assessed by amending growing media, respectively, with NaCl (Afzal et al., 2015), polyethylene glycol (PEG) (Kour et al., 2019), and the pollutant targeted for bioremediation (Iqbal et al., 2018), while plates may be incubated between 4 and 50°C to isolate psychrotolerant and thermotolerant bacteria, respectively (Kour et al., 2019). Screening conditions based on requirements for future commercialization steps can also advantageously pre-select bacteria that grow well on inexpensive media and in industrial fermenters used for mass production, that can withstand formulation processes such as freeze-drying, and that can thrive in the targeted cropping system in terms of temperature range, light intensity and compatibility with agrochemicals used (Köhl et al., 2011). Ultimately, the whole genome of selected strains can be sequenced and analyzed by *in silico* gene mining tools to provide a comprehensive overview of their potential beneficial traits, unveiling prospective bioactive compounds that would otherwise be overlooked by classical laboratory experimental methods and artificial conditions (Paterson et al., 2017).

### *In planta* Screening for Promising *Pseudomonas* spp.

Even though *in vitro* methods are appealing for high-throughput screening of microorganisms harboring beneficial traits, their shortcomings and biases are frequently pointed out, cautioning for example that nutrient-rich media and favorable growing conditions often do not reflect the complex natural environments in which the microorganisms should be active (Köhl et al., 2019). Accordingly, selecting the best *Pseudomonas* spp. performers during *in vitro* screening assays does not always guarantee success when subsequently inoculated on hemp plants (Balthazar et al., 2021). Inspiring innovations like leaf-based custom culture media (Elsawey et al., 2020) could help mimick cannabis intricate phyllosphere environment for rapid screening, while using cannabis hosts as “bait plants” or “recruiters” should also capture more host-adapted isolates from the soil (Zachow et al., 2013; Afzal et al., 2015; Ahmed and Hijri, 2021).

Additionally, many important bacterial modes of action cannot be properly assessed in bioassays without host plants, like ISR elicitation, rhizosphere colonization, microbiome interactions, phytohormonal and phytochemical modulations, plant growth promotion, and biocontrol activity against obligate biotrophic pathogens. In this regard, since processes like ISR elicitation (Balthazar et al., 2020), endosphere colonization (Winston et al., 2014; Comeau et al., 2020) and plant growth promotion (Afzal et al., 2015) seem to be genetically determined by host-microbe combinations in cannabis, as is generally the case in other plants (Bulgarelli et al., 2013; Pieterse et al., 2014), it would be important to start the screening process with the same cannabis cultivar as that intended for the end-use; as well as to use either seeds or clonal cuttings as intended later since this impacts the crop genetic uniformity. *In planta* bioassays can also reveal deleterious effects of the plant on the bacteria, such as antibacterial activity of cannabis phytochemicals (Ali et al., 2012), or, conversely, undesirable effects of the bacteria on the plant, such as HCN phytotoxicity (Trognitz et al., 2016), inhibition of root elongation at high IAA concentrations (Patten and Glick, 2002; Çakmakçı et al., 2020), reduced seed germination due to ACC deaminase activity (Nadeem et al., 2016), or altered sex determination of inflorescences due to phytohormonal disturbance (e.g., masculinization of female flowers or hermaphroditism, which is problematic in marijuana production where male flowers and pollination are undesirable) (Ganger et al., 2019; Punja and Holmes, 2020; Adal et al., 2021). These effects also illustrate that unilateral pre-screening of bacteria for higher secondary metabolite production and activity is not always a good strategy. Similarly, *Pseudomonas* strains selected for weed biocontrol should be tested for their selectivity against the targeted weeds and lack of damage to cannabis plants. If weed seeds are not readily available to screen for bioherbicidal effects, lettuce seeds are commonly used instead (Trognitz et al., 2016).

For all these reasons, it is advisable to introduce the intended cannabis host plant early in the screening process, under controlled conditions that would best represent the epidemiological and environmental realities of the crop, even if these large-scale experiments would require more time and resources (Köhl et al., 2011, 2019). Hopefully, high-throughput plant-based bioassays could benefit from recent innovations in cannabis biotechnological research, allowing for example the live visualization of root development and responses to elicitors (Suwanchaikasem et al., 2021), the automated estimation of hemp fiber yield and quality from scanner image analysis (Müssig and Amaducci, 2018), the high-resolution profiling of cannabinoids and plant extracts by chromatography coupled to mass spectrometry (Delgado-Povedano et al., 2020), the field-scale detection of diseases by drone remote sensing and machine learning (Bates, 2021), and the marker-assisted monitoring of cannabis pathways linked to pathogen defenses (Balthazar et al., 2020; McKernan et al., 2020; Pépin et al., 2021), abiotic stress responses (Mayer et al., 2015; Liu et al., 2016), phytochemical biosynthesis (Booth et al., 2017; Grassa et al., 2018; Jalali et al., 2019; Hesami et al., 2020), seed protein accumulation (Ponzoni et al., 2018), and fiber quality (Guerriero et al., 2017; Hesami et al., 2020).

### Consortia Versus Single-Strain Inoculants

Many studies in different crops have reported positive effects when combining several *Pseudomonas* strains together and/or with other microorganisms, as compared to single-strain inoculants. Beneficial microorganisms commonly used in consortia inoculants with *Pseudomonas* spp. include the bacterial genera *Bacillus*, *Rhizobium*, *Acinetobacter*, *Azospirillum*, and *Burkholderia*, and fungi *Glomus* and *Trichoderma* (Nadeem et al., 2016). Synergistic effects of such consortia are likely due to complementary modes of action and/or ecological requirements, resulting in more reliable functional outcomes than single organisms (Kaminsky et al., 2019). In this context, innovative holistic approaches have also been proposed, such as the design of synthetic microbial communities (SynComs, e.g., complex consortia mimicking microbiome functions, interaction networks and/or phylogenetic profiles) (Paredes et al., 2018; de Souza et al., 2020), core-microbiome therapy (e.g., transfer of an artificially cultivated microbiome from a healthy plant to a diseased one, like performed in clinical gastroenterology) (Gopal et al., 2013), prebiotic approaches (e.g., molecules stimulating the bioactivity of the resident microbiome and the growth of beneficial organisms) (Massart et al., 2015; Tabacchioni et al., 2021), or combination with helper strains (e.g., bacteria without beneficial properties by themselves but enhancing the efficacy of co-inoculated partners) (Massart et al., 2015; Berninger et al., 2018). In cannabis, several *Pseudomonas* strains in consortia with *Bacillus* strains promoted hemp growth significantly more than the corresponding single-strain inoculants (Comeau et al., 2021), but did not improve ISR elicitation against *Botrytis* gray mold disease (Balthazar et al., 2020). Other bacterial consortia also significantly promoted hemp growth and/or retting process, but comparisons with single-strain inoculants were not investigated (Di Candilo et al., 2010; Conant et al., 2017; Pagnani et al., 2018). Finally, two hemp endophytic *Pseudomonas* strains were found to be compatible for growth alongside fungal biocontrol agents *Trichoderma* and *Stachybotrys in vitro* (Scott et al., 2018), suggesting that they could be combined effectively in a joint bioproduct formulation. *In silico* screening (Tabacchioni et al., 2021), computer-assisted combination optimization (de Souza et al., 2020), and predictive association modeling strategies (Paredes et al., 2018) could also be implemented as preliminary steps to help with the rational design of custom microbial consortia for cannabis.

## Commercialization Bottlenecks and Challenges

Many traits contribute to the success of *Pseudomonas* spp. developed as commercial bioproducts, like their rapidity of growth and ease of mass production, their ability to readily use a wide array of nutrients and compete in the rhizosphere, their multiple modes of action and broad-spectrum activities, and their adaptability to a variety of plant-soil environments and stresses (Fischer et al., 2013).

In cannabis, the potential benefits of *Pseudomonas* spp. inoculants have already been demonstrated *in vitro* (Afzal et al., 2015; Iqbal et al., 2018; Scott et al., 2018; Balthazar et al., 2021) and in different cropping systems including greenhouses (Gonsior et al., 2004), hydroponic and soil-less systems (Conant et al., 2017), and indoor growth chambers (Balthazar et al., 2020, 2021; Comeau et al., 2021), while experiments in outdoor fields are still lacking. Notably, two of the aforementioned studies used commercially available *Pseudomonas*-based bioproducts (Gonsior et al., 2004; Conant et al., 2017).

A plethora of good reviews can be consulted for a general overview of commercialization steps, challenges, and bottlenecks commonly encountered when developing microbe-based bioproducts, including efficacy testing, risk assessments, formulation design, mass production, commercial registration, and marketing (Köhl et al., 2011; Backer et al., 2018; Kaminsky et al., 2019; Pirttilä et al., 2021). The following sections will therefore mainly focus on specific considerations when developing *Pseudomonas* spp. inoculants for cannabis crops (Figure 2).

### Risks for Crops and Human Health

As introduced above, *Pseudomonas* strains related to the species *P. syringae* and *P. aeruginosa* are common pathogens of plants and animals, including cannabis and humans, whereas plant-beneficial strains from the *P. fluorescens* group are generally considered safe for humans and crops (Holmes et al., 2015). Even though some *P. syringae* strains have been described as biocontrol agents and exhibit plant-beneficial attributes, the possibility of a switch to phytopathogenic behavior by horizontal gene transfer would make their introduction into crops extremely risky (Passera et al., 2019). Similarly, in some jurisdictions, *P. aeruginosa* is part of the blacklisted organisms and bile-tolerant Gram-negative (BTGN) bacteria that must be absent in marijuana products destined to human consumption (McPartland and McKernan, 2017; McKernan et al., 2018; Boyar, 2021), even though the need to test cured products has been questioned (Holmes et al., 2015). Therefore, to prevent any biosafety risk, bioproducts containing bacteria related to *P. syringae* or *P. aeruginosa* should not be used on cannabis crops. Consequently, proper taxonomic identification when screening candidate strains should be carried out with methods able to differentiate unambiguously between closely related *Pseudomonas* species, such as genome-wide comparisons or multilocus sequence analyzes (MLSA), rather than 16S ribosomal gene sequencing which mostly allows genera discrimination (Lalucat et al., 2020). The selected candidates can be further scrutinized to exclude potential producers of virulence factors and harmful toxins, based on whole-genome mining strategies and/or production detection assays (Balthazar et al., 2021). This step can help support their classification as safe organisms, thus facilitating the commercial registration process (Paterson et al., 2017). In this regard, beneficial *Pseudomonas* strains might have an advantage over mycotoxin-producing biocontrol agents like *Trichoderma* and *Stachybotrys* (Vujanovic et al., 2020), although the relevance of in-depth toxicological risk assessments of microbial metabolite residues on plants has been questioned, due to the low amounts produced *in planta* (Köhl et al., 2019) and subsequent heat degradation during marijuana product processing (Holmes et al., 2015). Besides, the common fear of introducing harmless microorganisms into edible plant parts has no real basis since plants tissues are already naturally colonized by such microorganisms and many healthy food products also contain microorganisms safe for human consumption (Pirttilä et al., 2021).

Unfortunately, even with carefully selected beneficial microorganisms, concerns about potential carry-over of contaminants from treated plants into medical or recreational products could still limit the use of bioproducts on drug-type crops. Indeed, commercial quality control procedures and regulatory requirements usually do not distinguish between beneficial and harmful microorganisms, which could lead to unwarranted rejection of the product (McKernan et al., 2016; Boyar, 2021; Punja and Ni, 2021). Restricting treatments to plant roots and vegetative parts, and/or long before harvest time, has been suggested in order to spare the valuable inflorescences from residues while still offering beneficial effects like plant growth promotion, abiotic stress tolerance and below-ground microbiome wealth (Conant et al., 2017; Pagnani et al., 2018; Comeau et al., 2021; Kakabouki et al., 2021a,b), as well as disease prevention by direct antibiosis against soilborne pathogens (Punja, 2021), and ISR elicitation (Balthazar et al., 2020) or sporulation suppression (Balthazar et al., 2021; Scott and Punja, 2021) against aerial pathogens. Additionally, more specific bacterial contaminant testing methods are urgently needed to resolve this important issue in cannabis cultivation, for example testing methods based on specific nucleic acid detection (McKernan et al., 2016, 2018, 2021; Boyar, 2021).

Surprisingly, the detection of fungal contaminants can also be compromised in the presence of chloramphenicol-resistant *P. fluorescens*, when culture-based methods that fail to prevent their proliferation are used (McKernan et al., 2016). Similarly, salicylic acid-producing *Pseudomonas* spp. can falsely elevate the total yeast and mold (TYM) count in marijuana products by interfering with pH-based detection methods (McKernan et al., 2018). These false-positive results may lead to undue product rejection and unnecessary fungicide applications. The necessary implementation of more accurate fungal detection methods, such as multiplex molecular assays, could therefore also play an important role in enabling the use of beneficial *Pseudomonas* spp. in marijuana cultivation (McKernan et al., 2016, 2021; Boyar, 2021).

### Risks for the Environment

Before microbial inoculants can be applied to cannabis crops, potential risks associated with non-target environmental effects should be examined (Mawarda et al., 2020), especially for outdoor crops. Indeed, in many countries, registration of microbial inoculants often require that they do not persist after a target time period, do not migrate off-site, nor have long-term impacts on natural communities. Such requirements can be in direct opposition to traits that were favored earlier in the screening process, like aggressive growth, dispersal, and competitiveness of microbial candidates, thus aggravating their invasive potential after large-scale release (Kaminsky et al., 2019). Approaches aiming to modify already established microbial communities would thus be ecologically sounder than methods aiming to replace them (Agoussar and Yergeau, 2021). Besides, plants under stress tend to mostly promote microorganisms that are already present in their environment, rather than recruiting new ones from applied inoculants (Giard-Laliberté et al., 2019). Survival and dispersal of *Pseudomonas* spp. are also expected to differ between indoor and outdoor cannabis cropping systems, with factors like weather, intercropping, crop debris, and natural competitors impacting open fields but not protected environments which are sanitized between growing cycles. In this context, non-sporulating *Pseudomonas* spp. might have an advantage over bacteria like *Bacillus* spp. whose stress-tolerant spores are particularly amenable to dry formulation and extended shelf-life but also promote undesired persistence in soil (Kaminsky et al., 2019). Environmental risks also depend on the mode of action exerted by the microorganisms (Köhl et al., 2019), with lower risks associated with mechanisms that do not require establishment and long-term survival of the bacteria, like ISR elicitation, early modulation of root architecture, or transient effects at a critical crop stage. However, disappearance of the inoculated strain does not necessarily imply a lack of lasting legacy on the native communities. In fact, the vast majority of studies report persistent changes in community composition as long-term consequences of microbial inoculation (Mawarda et al., 2020). To address these issues, it has been recommended to use native strains isolated from local ecosystems, and to appraise potential lasting impacts on microbiome composition, functioning and resilience with sensitive high throughput methods, for at least several months and/or growing seasons after application at multiple location sites (Köhl et al., 2011; Mawarda et al., 2020).

Finally, the possibility of an intensified spread of antibiotic resistance genes because of the introduction of beneficial bacteria into soils has been raised. Indeed, most PGPR strains, including common *Pseudomonas* spp. crop inoculants, have been found to be resistant to multiple antibiotics, likely because of inadvertent screening and/or co-selection with competitiveness abilities (Kang et al., 2017). While bacterial biofilms and plant-associated micro-environments are known hotspots for horizontal gene transfers (HGT) (Van Elsas et al., 2003), the occurrence of plasmid exchange from inoculated *P. fluorescens* to indigenous Gram-negative rhizobacteria in soil has been demonstrated under field conditions (van Elsas et al., 1998). Moreover, antibiotic-resistant microorganisms associated with edible crops may exacerbate the risk of dissemination and human exposure through the food chain and worldwide trade exchanges (Chen et al., 2019). To mitigate these issues, crop inoculants should neither contain bacteria resistant to multiple antibiotics or to important human drugs, nor contain genetically engineered strains marked with antibiotic resistance genes. Applications on crop leaves or seeds rather than by soil drench can also help mitigate the problem in soil, as would applications of bioactive metabolites instead of the living organisms that produced them (Kang et al., 2017).

### Formulations and Practical Applications

Developing bioproduct formulations that maintain high cell viability is an important bottleneck when working with non-sporulating Gram-negative bacteria like *Pseudomonas* spp. Attention should thus be given to inoculum concentration and product shelf-life. For commercial purposes, *Pseudomonas* spp. are usually mass-produced using liquid fermentation technologies and formulated as liquids, slurries, or solid forms (powders and granules) (Nakkeeran et al., 2005; Berninger et al., 2018). Since desiccation is often mainly responsible for the loss of viability of non-sporulating bacteria during bioproduct processing, storage, and field application, mitigation strategies are important to consider and include the validation of suitable drying methods, the addition of proper carriers and protectants (peat, talc, skimmed milk, etc.), and optional storage at cold temperature (Berninger et al., 2018). While most *Pseudomonas*-based bioproducts are commercialized at concentration superior to 10^6^ CFU/g with an average shelf-life of 6 months to a year (Nakkeeran et al., 2005; Berninger et al., 2018), cannabis plant growth can already be appreciably promoted at low inoculum density (Pagnani et al., 2018) and excessive concentrations could even lead to deleterious effects, as reported with other PGPR tested on cannabis (Pagnani et al., 2018; Comeau et al., 2021). However, considering the wide variety of cannabis production systems, formulating a range of bioproducts for reliable and consistent results under contrasting environments could be an exciting challenge. For example, the efficacy of inoculated bacteria will likely vary between outdoor and indoor crops, because of differential abiotic conditions (naturally fluctuating or controlled environments), exposure to adverse weather and rain (open fields or sheltered greenhouses and cultivation rooms), edaphic factors and resident microbiomes (natural soils or standardized growing substrates and soil-less hydroponic systems). The growing substrate alone was shown to substantially influence the outcome of synergistic interactions between *Pseudomonas* spp. and *Bacillus* spp. inoculants to promote cannabis growth and modulate resident microbiome in indoor cultivation (Comeau et al., 2021).

Compatibility with existing production practices and agrochemicals is another important hurdle when developing bioproducts for a broad market (Köhl et al., 2011). Agrochemicals applied simultaneously should not reduce *Pseudomonas* spp. viability, and the abilities of certain *Pseudomonas* spp. to degrade and/or reduce the effects of several herbicides, insecticides and fungicides—as detailed above—should also be considered (Kahlon, 2016; Nadeem et al., 2016; Trognitz et al., 2016). Furthermore, to ensure practical utilization by cannabis growers, formulations should be easy to apply with standard machinery and equipment and should be compatible with traditional cultivation techniques and organic certifications (Nakkeeran et al., 2005; Berninger et al., 2018). Thanks to their remarkable versatility, *Pseudomonas* spp. inoculants have been adapted to a variety of delivery methods commonly used in agriculture and horticulture, including seed coatings, soil amendments, root dips, foliar sprays, or multiple combination of these treatments (Nakkeeran et al., 2005; Berninger et al., 2018). Notably, in intensive cannabis cultivation systems, microbial inoculants can be circulated in irrigation water and hydroponic nutritive solution (Conant et al., 2017; Kakabouki et al., 2021a,b; Punja, 2021), while bacterial volatiles might be dispensed through atmosphere conditioning and ventilation infrastructures in greenhouses and indoor facilities (Garbeva and Weisskopf, 2020). Moreover, for drug-type crops reproduced by vegetative cuttings, shoot endophytic organisms inoculated on mother plants could be vertically transmitted to clonal cuttings (Comeau et al., 2020; Pirttilä et al., 2021). Similarly, for seeded oilseed and fiber crops, endophytic bacteria introduced into the inflorescences of parent plants could be transmitted inside the seeds to the offspring generation (Mitter et al., 2017; Berg and Raaijmakers, 2018). However, regulatory requirements for the marijuana market could substantially increase some production costs, such as the need to manufacture the bioproducts in sterile conditions and to use only food-safe additives to prevent marijuana product contaminations (Berninger et al., 2018; Pirttilä et al., 2021). In this regard, consortia-based products are also usually associated with additional research and production expenses compared to single-strain inoculants (Köhl et al., 2019), even though direct co-cultivation approaches exist for fully compatible *Pseudomonas* strains to spare the costly process of mass-producing each strain separately (Berninger et al., 2018).

Finally, guidelines for timing of application will need to be adapted according to crop-type, production goals, and bioproduct intended purpose. As discussed above, increased risk of microbial contaminants on marijuana products should be carefully examined when treating close to harvest (Punja, 2021). Application timing for PGPR strains that increase plant biomass should be aligned with phytochemical production goals (either maximizing inflorescence weight, or maximizing phytochemical concentration), as indicated by the distinctive effects of fertilizers when used at different cultivation stages—as explained above—(Caplan et al., 2017a,b; Saloner and Bernstein, 2021; Shiponi and Bernstein, 2021). Additionally, the use of biocontrol agents for preventive disease management should be based on forecasted risks for pathogen infestations and environmental factors (Balthazar et al., 2021; Punja and Ni, 2021). Multi-application schedules can also be advantageous for inoculants with transient effects which are preferable for their low environmental impacts (Kaminsky et al., 2019).

## Conclusion

The many different modes of action exerted by beneficial *Pseudomonas* spp. hold great potential to promote the yield and harvest quality of marijuana and hemp crops and could be exploited to target specific biotic and abiotic issues encountered by each crop type (grown for fibers, oilseeds, and/or phytochemicals). Traditional and innovative strategies to identify promising bacteria and formulate suitable bioproducts should provide useful avenues toward the development of effective inoculants.

## Author Contributions

CB: conceptualization, writing-original draft, and review and editing. DJ and MF: writing-review and editing and funding acquisition. All authors have read and approved the submitted version.

## Conflict of Interest

The authors declare that the research was conducted in the absence of any commercial or financial relationships that could be construed as a potential conflict of interest.

## Publisher’s Note

All claims expressed in this article are solely those of the authors and do not necessarily represent those of their affiliated organizations, or those of the publisher, the editors and the reviewers. Any product that may be evaluated in this article, or claim that may be made by its manufacturer, is not guaranteed or endorsed by the publisher.
